# Upregulation of Neuroinflammation-Associated Genes in the Brain of SARS-CoV-2-Infected Mice

**DOI:** 10.3390/pathogens13070528

**Published:** 2024-06-22

**Authors:** Soo-Jin Oh, Pratima Kumari, Tabassum Tasnim Auroni, Shannon Stone, Heather Pathak, Amany Elsharkawy, Janhavi Prasad Natekar, Ok Sarah Shin, Mukesh Kumar

**Affiliations:** 1 BK21 Graduate Program, Department of Biomedical Sciences, College of Medicine, Korea University Guro Hospital, Seoul 08308, Republic of Korea; sjooooh@gmail.com; 2 Department of Biology, College of Arts and Sciences, Georgia State University, Atlanta, GA 30303, USA; pratimakumari0306@gmail.com (P.K.); tauroni1@gsu.edu (T.T.A.); sstone12@student.gsu.edu (S.S.); hpathak1@student.gsu.edu (H.P.); aelsharkawy2@student.gsu.edu (A.E.); jnatekar1@student.gsu.edu (J.P.N.)

**Keywords:** SARS-CoV-2, COVID-19, neuroinflammation, TLR2 pathways, pyroptosis

## Abstract

Neurological manifestations are a significant complication of coronavirus disease 2019 (COVID-19), but the underlying mechanisms are yet to be understood. Recently, severe acute respiratory syndrome coronavirus 2 (SARS-CoV-2)-induced neuroinvasion and encephalitis were observed in K18-hACE2 mice, leading to mortality. Our goal in this study was to gain insights into the molecular pathogenesis of neurological manifestations in this mouse model. To analyze differentially expressed genes (DEGs) in the brains of mice following SARS-CoV-2 infection, we performed NanoString gene expression analysis using three individual animal samples at 1, 3, and 6 days post-infection. We identified the DEGs by comparing them to animals that were not infected with the virus. We found that genes upregulated at day 6 post-infection were mainly associated with Toll-like receptor (TLR) signaling, RIG-I-like receptor (RLR) signaling, and cell death pathways. However, downregulated genes were associated with neurodegeneration and synaptic signaling pathways. In correlation with gene expression profiles, a multiplexed immunoassay showed the upregulation of multiple cytokines and chemokines involved in inflammation and cell death in SARS-CoV-2-infected brains. Furthermore, the pathway analysis of DEGs indicated a possible link between TLR2-mediated signaling pathways and neuroinflammation, as well as pyroptosis and necroptosis in the brain. In conclusion, our work demonstrates neuroinflammation-associated gene expression profiles, which can provide key insight into the severe disease observed in COVID-19 patients.

## 1. Introduction

Since the first outbreak in China in 2019, coronavirus disease 2019 (COVID-19) has spread rapidly and globally, with a mortality rate of 2% resulting in an ongoing pandemic. The lack of highly efficacious antiviral drugs that can manage this ongoing global emergency increases the urgency of establishing a comprehensive understanding of the molecular pathogenesis of severe acute respiratory syndrome coronavirus 2 (SARS-CoV-2). Typical clinical presentations of COVID-19 can be characterized by mild or asymptomatic conditions; some patients experience more severe disease and develop systemic inflammation, tissue damage, acute respiratory distress syndrome, thromboembolic complications, cardiac injury, and/or cytokine storm [1,2]. Furthermore, SARS-CoV-2 infection is also associated with a wide variety of neurological manifestations, such as headache, loss of taste and smell, ataxia, meningitis, cognitive dysfunction, memory loss, seizures, and impaired consciousness, as well as long-term neurological problems in more than 30% of adults [3,4]. However, the mechanism by which SARS-CoV-2 infection causes neurological diseases remains unclear.

We previously showed that the intranasal infection of SARS-CoV-2 in K18-hACE2 mice resulted in brain encephalitis characterized by the secretion of cytokines and chemokines, leukocyte infiltration, hemorrhage, and neuronal cell death [5]. SARS-CoV-2 infects cells within the nasal turbinate, eye, and olfactory bulb, suggesting SARS-CoV-2’s entry into the brain by this route after intranasal infection. In addition, histopathological analyses revealed that neuroinflammation potentially resulted in the severe disease observed in SARS-CoV-2-infected K18-hACE2 mice. In a follow-up study, we demonstrated that the neuronal cultures obtained from K18-hACE2 mice are permissive to SARS-CoV-2 infection and support productive virus replication. Furthermore, SARS-CoV-2 infection upregulated the expression of genes involved in antiviral immunity and inflammation in the brain [6].

To further investigate the molecular pathogenesis of neurological manifestations in K18-hACE2 mice following SARS-CoV-2 infection, we profiled expression changes of approximately 786 selected genes covering major neuroinflammation pathways using the NanoString nCounter technology. Furthermore, we identified several significantly dysregulated and functionally interesting genes associated with neuroinflammation during SARS-CoV-2 infection. This study shed a new light on the molecular pathways and genes involved in neuroinflammation and cell death, which can help to develop targeted treatments and interventions for COVID-19 patients with neurological complications. 

## 2. Results

### 2.1. SARS-CoV-2 Infection of K18-hACE2 Mice Modulates Host Gene Expression in Brain Tissue

**Figure 1:**
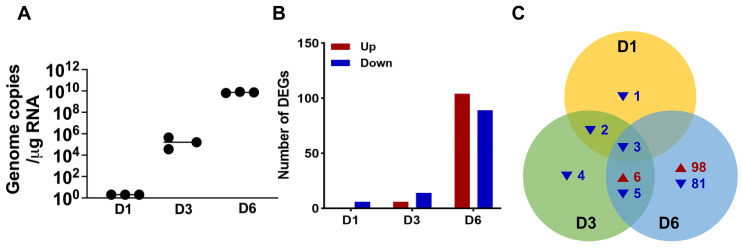
Gene expression profiles of SARS-CoV-2-infected brain in K18-hACE2 mice. (**A**) K18-hACE2 mice were intranasally infected with 10^5^ PFU SARS-CoV-2. At 1 (D1), 3 (D3), and 6 days (D6) post-infection, RNA was extracted from the brain, and qRT-PCR was performed to determine SARS-CoV-2 genome copies. Each dot represents one animal. (**B**) The graph shows the numbers of differentially expressed genes (DEGs) with more than 5-fold (Up) or less than −2-fold (Down) increases in expression compared to mock groups. (**C**) Venn diagram displays the number of overlapping DEGs from D1, D3, and D6 samples of SARS-CoV-2-infected K18-hACE2 brain. Red- and blue-colored numbers represent the upregulated and downregulated DEGs, respectively.

We previously established SARS-CoV-2 pathogenesis in a K18-hACE2 mouse model and demonstrated that intranasal inoculation with SARS-CoV-2 resulted in neuroinvasion and neurotropism [5,6]. To investigate the characteristics of SARS-CoV-2-induced immune response in the brain, K18-hACE2 mice were intranasally inoculated with SARS-CoV-2 (USA-WA1/2020), and brain tissues were collected on days 1, 3 and 6 post-infection. No significant weight loss was observed at day 1 and day 3 post-infection. As previously reported, infected animals started showing clinical symptoms of disease such as weight loss as well as neurological symptoms starting day 4 after the infection [5,6]. By 6 days, all mice infected with the virus lost between 10 and 15% body weight. We euthanized three individual animals at 1, 3, and 6 days post-infection. We euthanized mock control group animals on day 6. We identified the DEGs by comparing virus-infected animals with the mock control group animals. First, we confirmed the time-dependent increase in SARS-CoV-2 genome copies in the infected brains of K18-hACE2 mice (Figure 1A). Next, RNA was extracted from the brain tissues and analyzed using the NanoString nCounter technology to uncover gene expression patterns. We selected differentially expressed genes (DEGs) using the following criteria: fold change (FC) > 5 and <−2 were considered as the range for upregulated and downregulated genes, respectively. Figure 1B presents the number of DEGs at days 1, 3, and 6 post-SARS-CoV-2 infection. In total, 104 genes were significantly upregulated, and 89 genes were significantly downregulated at 6 days post-infection (D6), whereas there were no upregulated genes and only six downregulated genes at 1 day post-infection (D1). To show gene dysregulation, we generated a Venn diagram, and it showed 16 genes, including *Cxcl10* (encoding IP-10), *Ccl2* (encoding MCP-1), *Zbp1*, and *Irf7*, commonly dysregulated in response to SARS-CoV-2 infection (Figure 1C). Of note, most DEGs were detected only at D6, which correlated with high viral RNA in the brain and body weight loss. 

Table 1 summarizes the top 20 DEGs in the order of FC at each time point. Among upregulated genes, *Cxcl10* showed a 15-fold increase at day 3 post-infection (D3) and a 1300-fold increase at D6. Furthermore, cytokines and chemokines such as *Ccl2*, *Ccl5* (encoding RANTES), *Cxcl9* (encoding MIG), and *Ccl3* (encoding MIP-1*α*) showed an increase of more than 100-fold at D6. Among the downregulated genes, *Eomes*, *Fos*, and *Dxl1* were commonly downregulated in SARS-CoV-2-infected mice, and *Grm2*, *BDNF*, and *Gria4* were significantly downregulated at D3 and D6 (Table 1). 

### 2.2. Distinct Genes Regulated by SARS-CoV-2 Infection Show Activated Viral Sensing Pathways and Neurological Dysfunction in Brain

**Figure 2:**
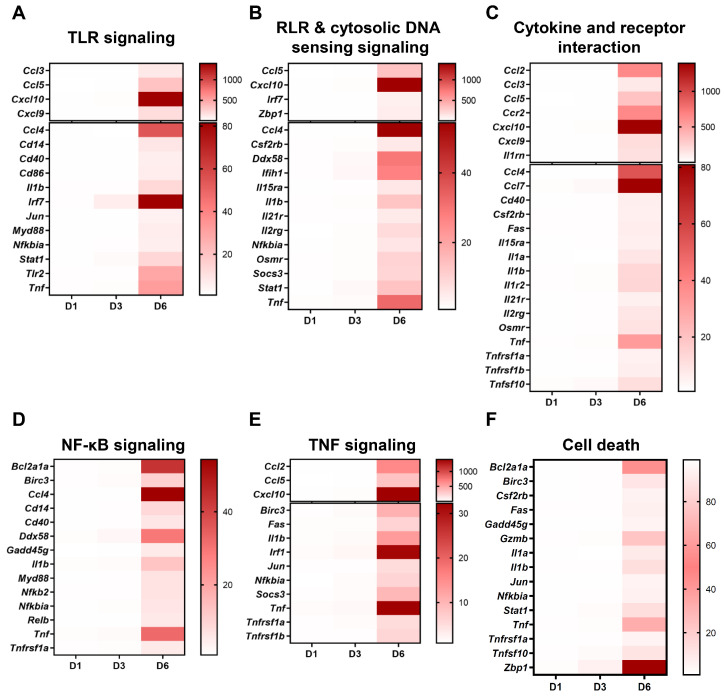
Upregulated genes in the brains of SARS-CoV-2-infected mice. Heatmaps showing the fold change in expression levels of specific genes involved in the following signaling pathways: (**A**) Toll-like receptor (TLR) signaling pathway (KEGG pathway: mmu04620), (**B**) RIG-I-like receptor (RLR) and cytosolic DNA sensing pathway (KEGG pathway: mmu04622 and mmu04623, respectively), (**C**) cytokine and receptor interaction (KEGG pathway: mmu04060), (**D**) nuclear factor kappa B (NF-kB) signaling pathway (KEGG pathway: mmu04064), (**E**) tumor necrosis factor (TNF) signaling pathway (KEGG pathway: mmu04668), and (**F**) cell death (KEGG pathway: mmu04210 and mmu04217). Red indicates the genes were upregulated in response to SARS-CoV-2 infection.

To examine which signaling pathways are involved in the expression of significant DEGs, we performed heatmap analysis (Figure 2). Notably, genes including *Ddx58* (encoding RIG-I), *Ifih1* (encoding MDA5), and *Tlr2*, involved in Toll-like receptor (TLR) and RIG-I-like receptor (RLR) signaling as well as genes associated with the cytosolic DNA sensing pathway were found to be highly upregulated (Figure 2A,B). In addition to the genes associated with pattern recognition receptor (PRR) signaling, genes closely linked with nuclear factor kappa B (NF-κB) and tumor necrosis factor (TNF) signaling pathways were also found to be upregulated at D6 (Figure 2D,E). Furthermore, in terms of the NF-κB signaling pathway, transcriptional factors involved in the canonical pathway, *Cd40* (encoding p50) and *Nfkb2* (encoding p100), and transcriptional factors involved in the non-canonical pathway, *Nfkbia* (encoding IκBα) and *Relb*, were activated [7]. We previously reported the activation of the inflammatory response in the brain [6]. Here, we examine the genes responsible for cytokine and chemokine production. Numerous genes encoding the CCL family, such as *Ccl2*, *Ccl3*, and *Ccl5*; the CXCL family, such as *Cxcl9* and *Cxcl10*; the TNF family, such as *tnfsf10* and *tnfrsf1b*; and the interleukin (IL) family, such as *Il1a*, and *Il1b* were significantly upregulated in the brain after SARS-CoV-2 infection (Figure 2C). Notably, the expression of *Cxcl10*, *Tnf*, *Il1a*, and *Il1b*, the final products of the PRR signaling pathway, increased by 20–40-fold compared to mock groups. 

**Figure 3:**
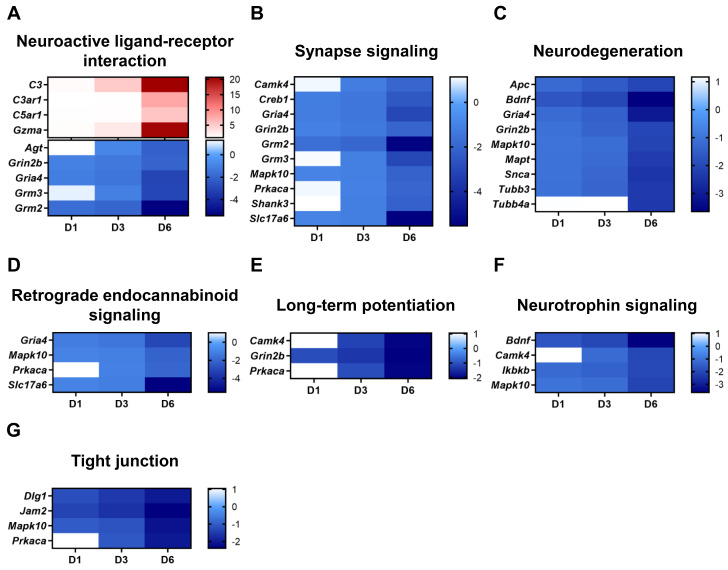
Neuronal function-regulated gene expression in the brains of SARS-CoV-2-infected mice. Fold change in the expression of nervous system-related genes represented by heatmaps. (**A**) Neuroactive ligand–receptor interaction (KEGG pathway: mmu04080), (**B**) synapse signaling pathway (KEGG pathway: mmu04724, mmu04727, mmu04725, mmu04728, and mmu04726), (**C**) neurodegeneration (KEGG pathway: mmu05022), (**D**) retrograde endocannabinoid signaling (KEGG pathway: mmu04723), (**E**) long-term potentiation (KEGG pathway: mmu04720), (**F**) neurotrophin signaling (KEGG pathway: mmu04722), and (**G**) tight junction (KEGG pathway: mmu04530) are shown. Red and blue boxes indicate the upregulation and downregulation of specific genes, respectively.

Downregulated genes were also analyzed. Surprisingly, several genes that play major roles in synapse signaling and neuronal functions, such as the retrograde endocannabinoid, long-term potentiation, and neurotrophin signaling pathways, were downregulated by SARS-CoV-2 (Figure 3). In detail, synapse signaling pathways can be divided into five categories, as follows: gamma-aminobutyric acidergic, cholinergic, dopaminergic, serotonergic, and glutamatergic synapse signaling [8]. Among these synapse signaling pathways, most of the downregulated genes contribute to glutamatergic synapse signaling, such as *Gria4*, *Grin2b*, *Grm2*, and *Grm3* (Figure 3B). We also evaluated the genes associated with tight junction proteins to see if SARS-CoV-2 infection impaired tissue integrity and enhanced brain–blood barrier permeability. We detected four genes, *Jam2*, *Prkaca*, *Dig1*, and *Mapk10,* that were involved in tight junction and cellular adhesion mechanisms and were downregulated (Figure 3G). 

**Figure 4:**
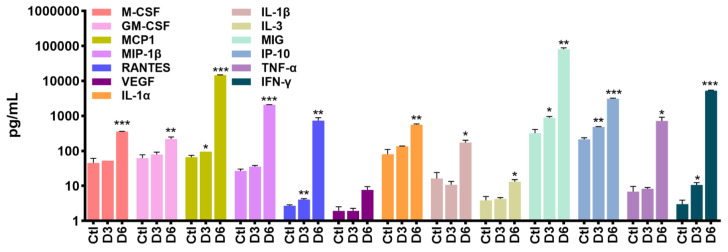
Cytokine and chemokine levels are differentially expressed in the brains of SARS-CoV-2-infected mice. To measure cytokine secretion levels, brain homogenates from mock- (Ctl) and SARS-CoV-2-inoculated mice were collected at days 3 (D3) and 6 (D6) after infection. Cytokine secretion levels in brain homogenates were examined via MILLIPLEX using the manufacturer’s instructions. Five-parameter logistics of the spline curve fitting method were used as the standard curve, and data were calculated using MILLIPLEX Analyst software 5.1. Each point represents the mean ± SD. Statistical analysis: * *p* < 0.05, ** *p* < 0.01, *** *p* < 0.001, compared to mock control groups.

To validate gene expression profiling, we performed a Luminex assay on brain homogenates and measured the protein level of cytokines and chemokines. We used three individual animals at 1, 3, and 6 days post-infection. We also used three mock control group animals. Correlating with NanoString data, the pro-inflammatory cytokines IL-1α, IL-1β, TNF- α, and IFN-γ were significantly increased compared to the mock control at D3 and D6 (Figure 4). 

### 2.3. SARS-CoV-2 Infection Causes Neuro-Inflammatory Response Following Antiviral Response Activation

To characterize the molecular function and biological process of DEGs, we performed gene ontology (GO) and Kyoto Encyclopedia of Genes and Genomes (KEGG) analyses. GO analysis showed that diverse immune responses, such as defense response to virus, immune system processes, and innate immune response were activated at D3 and persisted through to D6 (Table 2). Along with the immune system, responses like the chemokine-mediated signaling pathway, the killing of cells of other organisms, and neutrophil chemotaxis slightly increased at D3. However, multiple biological pathways involving the inflammatory response, innate immune response, and cellular response to lipopolysaccharides were strongly increased at D6. In terms of molecular function, protein binding, chemokine activity, and cytokine activity were detected at both D3 and D6. 

Correlating with GO analysis, the KEGG pathways showed the sequential activation of innate immune and inflammatory responses, followed by cytokine and chemokine production and signaling pathways (Table 3). At D3, PRR signaling pathways like cytosolic DNA sensing, RIG-I-like receptor signaling, and Toll-like receptor signaling dominated the pro-inflammatory cytokine production-related signaling pathways, like TNF signaling, and several virus pathways, including coronavirus disease—COVID-19. At day 6, the pro-inflammatory cytokine production pathways, such as cytokine–cytokine receptor interaction and TNF signaling, were ranked high, followed by virus pathways such as coronavirus disease—COVID-19. Overall, the number of genes involved in the detected KEGG pathways increased on D6 to about 20–25 compared to D3. 

### 2.4. TLR Signaling and Programmed Cell Death Pathways Are Strongly Induced in SARS-CoV-2-Infected Brains 

**Figure 5:**
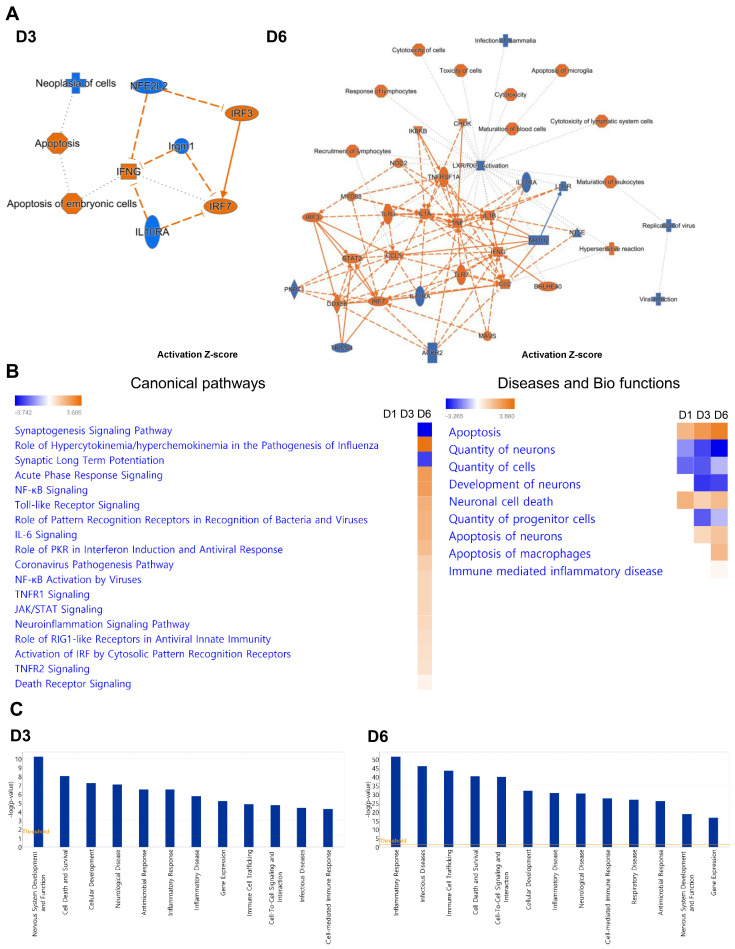
Top canonical pathways activated in the SARS-CoV-2-infected brain. (**A**) Graphical abstracts were generated for the overall changes in gene expression patterns using ingenuity pathway analysis (IPA) at days 3 (D3) and 6 (D6) after infection. (**B**) Comparative canonical pathways and (**C**) individual analyses for disease and disorders in response to SARS-CoV-2 infection were generated using IPA tools. The orange line indicates the default *p* value significance threshold of 0.05.

Next, ingenuity pathway analysis (IPA) was performed to delineate the SARS-CoV-2-mediated activation of neuro-inflammatory pathways in depth. Figure 5A shows graphical summaries of the brains of SARS-CoV-2-infected mice. *IRF3* and *IRF7* were elevated at both D3 and D6. In addition, *IFNG*, *IL1A*, and *IL1B* showed substantial crosstalk between TLR signaling pathway mediators such as *TLR3*, *TLR7*, and *MYD88* as well as key molecules of innate immune responses such as *DDX58*, *MAVS*, *IRF3*, and *IRF7*. 

Correlating with heatmaps and KEGG pathway analyses, comparative analyses for canonical pathways showed that Toll-like receptor signaling, the role of pattern recognition receptors in recognition bacteria and viruses, the role of RIG-I-like receptors in antiviral innate immunity, and activation of IRF by cytosolic pattern recognition receptors were activated in response to SARS-CoV-2 infection in the brain, in order of significance (Figure 5B). Specifically, several pathways related to cytokine and chemokine production pathways, such as the role of hypercytokinemia/hyperchemokinemia in the pathogenesis of influenza, NF-kB signaling, and the TNFR signaling pathway, were detectable at D6, in sequence. We also found that the synaptogenesis signaling pathway and synaptic long term potentiation were significantly downregulated. However, neurological complications such as the coronavirus pathogenesis pathway and neuroinflammation signaling pathway were increased. In addition to neuronal function alterations, the quantity of neurons, cells, and progenitor cells as well as the development of neurons were decreased in response to SARS-CoV-2 infection. In contrast, apoptosis, the apoptosis of neurons, and neuronal cell death were elevated. Furthermore, individual analyses for disease and disorders highlighted that neurological diseases are associated with SARS-CoV-2 infection (Figure 5C). Nervous system development and function, neurological disease, inflammatory disease, and infectious disease were significantly disrupted, as were antimicrobial response and inflammatory responses at D3. However, inflammatory responses and inflammatory disease were increased at D6, suggesting that inflammation in the brain tissue accelerated as SARS-CoV-2 infection progressed.

**Figure 6:**
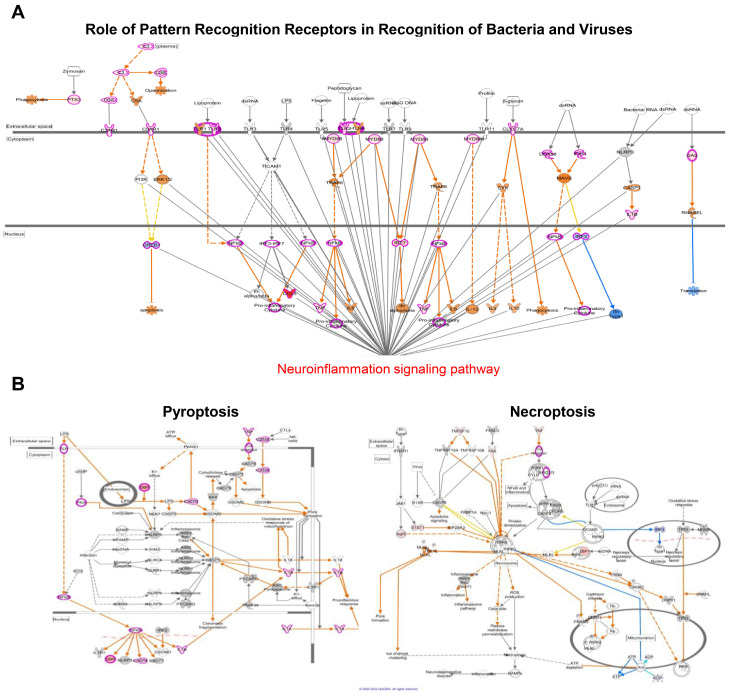
Identification of top canonical pathways activated by SARS-CoV-2-infected brains. Network maps show the pathways regulated by SARS-CoV-2 infection. At 6 days post-infection, the gene expression related to (**A**) PRR signaling merged with the neuroinflammation signaling pathway, while (**B**) pyroptosis and necroptosis are represented using the ingenuity pathway analysis (IPA) tool.

To explore the mechanisms behind SARS-CoV-2-induced neuroinflammation, we generated network maps with a significantly increased z-score. At D6, TLR1/2 and TLR2/6 heterodimers and their downstream mediators MYD88 and TRAF6 were highly upregulated, and these are linked with NF-kB- and IRF-dependent cytokine production pathways (Figure 6A). Together with the PRR signaling pathway, we identified several genes at D6 related to cell death pathways such as pyroptosis, necroptosis, and apoptosis (Figure 2F). As shown by our previous report, cell death pathways such as pyroptosis and necroptosis are more activated than apoptosis in response to SARS-CoV-2 infection. These pathways are related to upstream inflammation triggered by IL-1β secretion (Figure 4 and Figure 6B) [6]. 

## 3. Discussion

Multiple research groups have suggested the possibility of neurotropism and neurological pathology caused by SARS-CoV-2 [2,9,10,11,12]. Additionally, we and others have reported that the inoculation of SARS-CoV-2 in K18-hACE2 mice resulted in neuroinvasion and neurological diseases [5,13]. In the present study, we explored the molecular underpinnings of COVID-19 neurological complications using SARS-CoV-2-infected mice. First, we found that genes linked to TLR signaling and cell death pathways were upregulated, while genes associated with neurodegeneration and synaptic signaling were downregulated. We also identified the potential role of TLR2 in SARS-CoV-2-induced neuroinflammation and its potential link to other neurodegenerative diseases. Furthermore, the study’s insights into the imbalance in synapse signaling and synaptic dysfunction provide crucial information for investigating potential long-term neurological consequences in COVID-19 survivors.

Our comprehensive gene expression analysis indicates the specific induction of RLR and TLR signaling pathways, as shown by heatmaps (Figure 2A,B). In addition, the key mediators involved in NF-κB, TNF, and JAK/STAT signaling were also increased by SARS-CoV-2 infection, consistent with previous publications [14,15,16]. In addition to RLR and NLR, the essential role of TLRs was highlighted. Accumulating evidence suggests the involvement of TLRs in neurodegenerative diseases. TLRs are typically expressed in neurons, astrocytes, and microglia. In detail, microglia express all TLRs, whereas resting astrocytes express low levels of TLR2, 4, 5, and 9 and activate astrocytes expressing TLR2 [17,18,19,20]. During SARS-CoV-2 infection, the TLR2/MyD88 signaling pathway can detect envelope and spike proteins of SARS-CoV-2, and these are linked with NF-κB-mediated inflammatory responses [16,21,22]. In our study, genes involved in TLR2/MyD88 signaling pathways were activated by SARS-CoV-2 infection, and pro-inflammatory cytokines like IL-1α, IL-1β, TNF-α, and IFN-γ were increased in the brain (Figure 4 and Figure 6A). Considering our data, TLR2 may affect neuroinflammation and neuronal cell death. Moreover, it is well known that TLR2 accelerates the pathology of neurological disorders such as Alzheimer’s disease (AD), Parkinson’s disease (PD), and neuroinflammation. Relevant to this information, the blockage of TLR2–MyD88 interaction prevented neuroinflammation and attenuated AD’s pathology [23,24]. Therefore, further studies are needed to examine whether TLR2 directly induces neuroinflammation and cell death at cellular levels. 

It has been reported that SARS-CoV-2 infection activates various forms of cell death, including pyroptosis, apoptosis, and necroptosis [25,26,27,28]. We previously reported that SARS-CoV-2 infection activated necroptosis via ZBP1, RIPK1/3, and MLKL, with possible extrinsic apoptosis or pyroptosis activation, as shown by the increase in the expression of caspases [6]. Since pyroptosis is closely linked with inflammation by the cleavage of cytokine precursors and the activation of microglia or astrocytes, it could possibly contribute to neuroinflammation [29,30,31]. Necroptosis is distinguished from pyroptosis as RIPK1 and RIPK3 mediate them; however, both induce cell lysis and release damage-associated molecular patterns (DAMPs) to cause inflammation [32]. In CNS, both RIPK1 and RIPK3 are implicated in playing a role in neurological diseases such as multiple sclerosis, amyotrophic lateral sclerosis (ALS), AD, and PD by promoting neuroinflammation, cytokine production by microglia, and astrocyte and neuronal cell death [33,34,35,36]. Our IPA data demonstrate the activation of pyroptosis and necroptosis by SARS-CoV-2 as well as the crosstalk between these cell death pathways and IL-1α and IL-1β production (Figure 4 and Figure 6B). Additionally, as previously mentioned, there is a four-fold increase in caspase expression, including Casp1, Casp7, and Casp8, in comparison to the mock control (Figure 2F). ZBP1 expression is implicated in inflammatory cell death. Since SARS-CoV-2 infection is closely associated with both cell death and inflammatory responses, more research is needed to understand how the virus triggers various cell death pathways and triggers inflammatory responses. 

It should also be noted that genes involved in synaptogenesis signaling and long-term synaptic potentiation were differentially expressed, as detected by comparative IPA analysis for canonical pathways (Figure 5B). Importantly, we observed that the genes involved in neuroactive ligand–receptor interaction and synapse signaling pathways were significantly downregulated (Figure 3A,B). Neurotransmitters that can be divided into excitatory and inhibitory neurotransmitters are used in synaptic signaling pathways. Functionally, GABA, glycine, and serotonin are the main inhibitory neurotransmitters, while glutamate, aspartate, dopamine, epinephrine, and norepinephrine are considered excitatory neurotransmitters [8]. The balance of excitatory and inhibitory synapse signaling must be tightly regulated and maintained for synapse plasticity and the efficient functioning of signal transduction. Otherwise, an imbalance in synapse signaling might result in neurological complications such as AD, PD, and ALS [37]. According to our data, most DEGs related to synapse signaling are involved in excitatory neurotransmitters, especially glutamatergic excitatory synapse signaling, which mediates four of the major glutamic receptors: N-methyl-D-aspartate receptors that consist of the Grin family, AMPA receptors that consist of the Gria family, Kaniate receptors that consist of the Grik family, and metabotropic glutamate receptors that consist of the Grm family [38]. In particular, several receptors belonging to the Gria, Grin, and Grm families were significantly suppressed in SARS-CoV-2-infected mouse brains, suggesting that SARS-CoV-2 induces an imbalance in synapse signaling and synaptic dysfunction. Together with alterations in synapse signaling, several genes involved in retrograde endocannabinoid signaling, long-term potentiation, and neurotrophin signaling were slightly downregulated (Figure 3D–F). One of the limitations of this study is that we did not compare SARS-CoV-2-induced transcriptional changes with other stimuli such as LPS. Another limitation includes the use of a mouse model of severe COVID-19. Additionally, there is need for further clinical evidence to establish a direct link between these molecular changes and human neurological complications

In conclusion, our study has expanded the role of neuroinflammation-related genes during SARS-CoV-2 infection in neurological complications. In SARS-CoV-2-infected brains, the gene expression patterns were significantly disrupted, resulting in the robust activation of the immune response and inflammation. It is possible that the activation of the TLR signaling pathway, especially TLR2, may contribute to inflammation via NF-κB and TNF signaling pathways and programmed cell death, such as pyroptosis, in the brain. As novel variants of SARS-CoV-2 emerge and threaten public health, studies of the influence of SARS-CoV-2 variants are needed and can provide important insight into SARS-CoV-2 neuropathogenesis. Also, it would be interesting to test whether TLR2-targeting drugs can mitigate SARS-CoV-2-induced neuroinflammation and have any potential in therapeutic application. 

## 4. Materials and Methods

### 4.1. SARS-CoV-2 Infection in Mice

This study was performed following the National Institutes of Health and the Institutional Animal Care and Use Committee (IACUC) guidelines. The protocol was approved by the Georgia State University IACUC (protocol number A20044). To infect mice with SARS-CoV-2, we performed all the animal experiments in the Animal Biosafety Level 3 laboratory (ABSL3). Mice that met the human endpoint criteria were euthanized to limit suffering. As previously described, SARS-CoV-2 (USA-WA1/2020) was isolated from an oropharyngeal swab from a patient in Washington, USA (BEI NR-52281) [39]. Virus titration was performed using VeroE6 cells [5,39]. Hemizygous K18-hACE2 mice were obtained from the Jackson Laboratory (Bar Harbor, ME) [5]. Six-week-old K18-hACE2 mice were transferred to the ABSL-3 facility and acclimated to the local surroundings before initiating experiments. Nine animals were intranasally inoculated with 10^5^ PFU of SARS-CoV-2 (USA-WA1/2020), whereas three animals (mock control group) were inoculated with equivalent amounts of PBS [5]. Approximately similar numbers of male and female mice were used. During experiments, mice were checked for body weight, appetite, activity, and neurological signs every day following SARS-CoV-2 infection. On days 1, 3, and 6 after inoculation, the mice were euthanized to collect brain tissues. We euthanized three individual animals at 1, 3, and 6 days post-infection. We euthanized mock control group animals on day 6. We identified the DEGs by comparing virus-infected animals with the mock control group animals. The mock and SARS-CoV-2-infected mice were anesthetized using isoflurane and perfused with PBS. Later, RNA was collected from the brain. 

### 4.2. RNA Extraction and Evaluation

Frozen brain tissues harvested from mock (n = 3) and infected animals (n = 9) were weighed and lysed in RLT buffer (Qiagen), and RNA was extracted using an RNeasy MiniKit (Qiagen, Hilden, Germany) using the manufacturer’s instructions [40]. To measure the purity and quantity of total RNA, a Bioanalyzer 2100 (Agilent Technologies, Santa Clara, CA, USA) and NanoDrop spectrophotometer (Thermo Fisher Scientific, Waltham, MA, USA) were used. The following criteria were maintained: wavelength absorbance ratio A260/280 ~2.0 and A260/230 ~2.0. The percentage of RNA fragments ≥300 and nucleotides DV300 was ≥50%, and the integrity RIN was >4.

### 4.3. Quantification of the Virus Load

Quantitative RT-PCR was used to measure viral RNA levels with primers and probes specific for the SARS-CoV-2 N gene. Total RNA extracted from the tissues was quantified and normalized. Viral RNA levels per μg of total RNA were calculated using a standard curve generated using a known amount of RNA extracted from previously titrated SARS-CoV-2 samples [5].

### 4.4. NanoString nCounter^®^ Gene Expression

A commercially available NanoString nCounter^®^ Mouse Neuroinflammation Profiling Panel was used to count 786 immune-related genes (NanoString, Cat: XT-CSO-MIP1-12) following the NanoString guidelines. A set of housekeeping genes was used to normalize gene expression using the nSolver Analysis Software 4.0 (NanoString) [41]. At all time points, transcript quantification was performed for each SARS-CoV-2-infected mouse and mock-infected controls. We used three individual animals at 1, 3, and 6 days post-infection. We also used three mock control group animals. There was minimal animal-to-animal variation in gene expression patterns. The generated transcript average of each group was compared to the average of the mock-infected animals to determine the number of significantly differentially expressed genes. 

### 4.5. Luminex Measurement of Cytokines and Chemokines

The brains from mock and SARS-CoV-2-infected mice were homogenized and centrifuged at 13,000 rpm for 10 min, as previously reported [41]. Cytokine levels (GM-CSF, IFN-γ, IL-1α, IL-1β, IL-3, IP-10, MCP-1, M-CSF, MIG, MIP-1β, RANTES, TNF-α, and VEGF) were measured using a MILLIPLEX Mouse Cytokine/Chemokine Magnetic Bead Panel (Millipore, Norcross, GA, USA). Five-parameter logistics of the spline curve-fitting method standard curves were generated, and the samples’ concentrations of cytokines were calculated using MILLIPLEX Analyst 5.1 software (Millipore). 

### 4.6. Gene Ontology (GO) and Pathway Enrichment Analysis Using Kyoto Encyclopedia of Genes and Genomes (KEGG)

GO (david.ncifcrf.gov/) and KEGG (www.genome.jp/kegg/pathway.html) pathway analyses were conducted to identify DEGs at the biologically functional level [42]. Among the GO classification, the three categories of biological process, cellular component, and molecular function as well as the number of involved genes were represented. 

### 4.7. Ingenuity Pathway Analysis (IPA)

NanoString data were analyzed using IPA (QIAGEN, Redwood City, CA, USA), as described previously [41,43,44]. Briefly, graphical abstracts, comparative analyses, and individual analyses were undertaken using the list of DEGs identified by NanoString and the IPA Knowledge Base. The Ingenuity Knowledge Base is the largest database of manually curated and experimentally validated physical, transcriptional, and enzymatic molecular interactions. Statistical comparisons between pathways and network data, *p* values (Fisher’s exact test), and activation z-scores were calculated by IPA. A *p* < 0.05 was considered significant.

### 4.8. Statistical Analysis

For Nanostring, we used three individual animals at 1, 3, and 6 days post-infection. We also used three mock control group animals. The generated transcript average of each group was compared to the average of the mock-infected animals to determine the number of significantly differentially expressed genes. Differentially expressed genes (DEGs) were determined using the following criteria: fold change (FC) > 5 and <−2 as the range for upregulated and downregulated genes, respectively. Cytokine secretion levels in brain homogenates were examined via MILLIPLEX. The five-parameter logistics of the spline curve-fitting method was used for the standard curve, and data were calculated by using MILLIPLEX Analyst software 5.1. Data are presented as the mean ± standard deviation (SD). Statistical comparisons between different treatments were performed using Mann–Whitney U tests or Student’s *t*-tests, and the results were considered statistically significant at *p* < 0.05.

## Figures and Tables

**Table 1:** List of top up- and downregulated genes in the brains of SARS-CoV-2-infected K18-hACE2 mice at 1, 3, and 6 days post-infection. FC = fold change.

Upregulated Genes						Downregulated Genes					
D1		D3		D6		D1		D3		D6	
Gene	FC	Gene	FC	Gene	FC	Gene	FC	Gene	FC	Gene	FC
		*Cxcl10*	15.27	*Cxcl10*	1393.91	*Eomes*	−9.00	*Eomes*	−14.51	*Gpr34*	−10.18
		*Lcn2*	9.45	*Ccl2*	651.09	*Arc*	−3.12	*Dlx1*	−4.10	*Eomes*	−7.81
		*Irf7*	6.29	*Lcn2*	426.24	*Dlx1*	−2.97	*Arc*	−3.92	*Ttr*	−7.23
		*Zbp1*	6.11	*Ccl5*	328.46	*Cdkn1a*	−2.78	*Reln*	−2.76	*Cdc7*	−5.84
		*Ccl2*	5.89	*Rsad2*	219.17	*Dlx2*	−2.58	*Dlx2*	−2.53	*Slc17a6*	−5.55
		*Rsad2*	5.83	*Cxcl9*	186.42	*Fos*	−2.05	*Map3k1*	−2.30	*Grm2*	−5.44
				*Il1rn*	167.77			*Homer1*	−2.27	*Cx3cr1*	−4.45
				*Gbp2*	134.63			*Ago4*	−2.24	*Sall1*	−4.20
				*Ccl3*	126.66			*Sox4*	−2.23	*Ugt8a*	−4.19
				*Zbp1*	99.06			*Fos*	−2.09	*Chn2*	−4.06
				*Irf7*	81.45			*Dot1l*	−2.07	*Fcrls*	−4.04
				*Ccl7*	80.87			*Bdnf*	−2.05	*P2ry12*	−3.90
				*Lilrb4a*	55.60			*Sall1*	−2.04	*Dlx1*	−3.79
				*Ccl4*	54.35			*Grm2*	−2.01	*Slc2a5*	−3.67
				*Psmb8*	53.35					*Bdnf*	−3.66
				*Bcl2a1a*	43.68					*Adamts16*	−3.41
				*Sell*	39.69					*Stmn1*	−3.30
				*Casp4*	35.66					*Ago4*	−3.25
				*Ptx3*	35.53					*Gria4*	−3.12
				*Slfn8*	34.66					*Reln*	−3.09

**Table 2:** Gene ontology (GO) analysis of brains of SARS-CoV-2-infected mice.

Category	GO ID	Term	# Genes (%)	*p* Value
**Day 3**				
Biological process	GO:0051607	defense response to virus	4 (66.67%)	1.52 × 10^−5^
	GO:0002376	immune system process	4 (66.67%)	1.62 × 10^−4^
	GO:0045087	innate immune response	4 (66.67%)	3.98 × 10^−4^
	GO:0009617	response to bacterium	3 (50.00%)	2.01 × 10^−3^
	GO:0060340	positive regulation of type I interferon-mediated signaling pathway	2 (33.33%)	0.005811579
	GO:0090026	positive regulation of monocyte chemotaxis	2 (33.33%)	0.005822179
	GO:0070098	chemokine-mediated signaling pathway	2 (33.33%)	0.012371086
	GO:0031640	killing of cells of other organism	2 (33.33%)	0.018885435
	GO:0030593	neutrophil chemotaxis	2 (33.33%)	0.020134244
Cellular component	GO:0005576	extracellular region	3 (50.00%)	0.065899318
	GO:0005615	extracellular space	3 (50.00%)	0.072143421
Molecular function	GO:0005515	protein binding	5 (83.33%)	0.027720426
	GO:0008009	chemokine activity	2 (33.33%)	0.011893042
	GO:0008201	heparin binding	2 (33.33%)	0.046110545
	GO:0005125	cytokine activity	2 (33.33%)	0.061699185
**Day 6**				
Biological process	GO:0002376	immune system process	28 (27.18%)	1.01 × 10^−20^
	GO:0006954	inflammatory response	26 (25.24%)	2.05 × 10^−21^
	GO:0045087	innate immune response	23 (22.33%)	2.19 × 10^−12^
	GO:0006955	immune response	21 (20.39%)	1.52 × 10^−13^
	GO:0045944	positive regulation of transcription from RNA polymerase II promoter	19 (18.45%)	2.68 × 10^−5^
	GO:0051607	defense response to virus	18 (17.48%)	1.45 × 10^−15^
	GO:0071222	cellular response to lipopolysaccharide	17 (16.50%)	1.40 × 10^−12^
	GO:0010628	positive regulation of gene expression	17 (16.50%)	2.75 × 10^−8^
	GO:0032760	positive regulation of tumor necrosis factor production	16 (15.53%)	2.14 × 10^−17^
	GO:0009617	response to bacterium	15 (14.56%)	1.34 × 10^−10^
Cellular component	GO:0016020	membrane	52 (50.49%)	1.54 × 10^−4^
	GO:0016021	integral component of membrane	48 (46.60%)	0.004153123
	GO:0005886	plasma membrane	46 (44.66%)	2.11 × 10^−4^
	GO:0005737	cytoplasm	45 (43.69%)	0.047232
	GO:0005576	extracellular region	36 (34.95%)	8.19 × 10^−13^
	GO:0005615	extracellular space	31 (30.10%)	7.79 × 10^−9^
	GO:0005829	cytosol	31 (30.10%)	0.011572543
	GO:0009897	external side of plasma membrane	27 (26.21%)	1.29 × 10^−16^
	GO:0009986	cell surface	25 (24.27%)	2.53 × 10^−13^
	GO:0005887	integral component of plasma membrane	13 (12.62%)	0.02582786
Molecular function	GO:0005515	protein binding	48 (46.60%)	1.65 × 10^−5^
	GO:0042802	identical protein binding	31 (30.10%)	2.21 × 10^−8^
	GO:0005125	cytokine activity	13 (12.62%)	2.43 × 10^−9^
	GO:0003700	transcription factor activity, sequence-specific DNA binding	9 (8.74%)	0.0153461
	GO:0042803	protein homodimerization activity	9 (8.74%)	0.053333585
	GO:0004888	transmembrane signaling receptor activity	8 (7.77%)	1.38 × 10^−4^
	GO:0008009	chemokine activity	7 (6.80%)	9.10 × 10^−8^
	GO:0002020	protease binding	7 (6.80%)	1.70 × 10^−4^
	GO:0019899	enzyme binding	7 (6.80%)	0.036582315
	GO:0008233	peptidase activity	7 (6.80%)	0.048234665

**Table 3:** KEGG analysis of brains of SARS-CoV-2-infected mice.

Sample	Entry	KEGG Pathway	# Genes
Day 1	mmu04921	Oxytocin signaling pathway	2
Day 3	mmu04657	IL-17 signaling pathway	4
	mmu05171	Coronavirus disease—COVID-19	3
	mmu04623	Cytosolic DNA-sensing pathway	3
	mmu04622	RIG-I-like receptor signaling pathway	3
	mmu04668	TNF signaling pathway	3
	mmu04620	Toll-like receptor signaling pathway	3
	mmu04010	MAPK signaling pathway	3
Day 6	mmu04060	Cytokine–cytokine receptor interaction	25
	mmu04010	MAPK signaling pathway	21
	mmu05171	Coronavirus disease—COVID-19	20
	mmu04620	Toll-like receptor signaling pathway	20
	mmu04668	TNF signaling pathway	18

## Data Availability

Data are contained within the article.
